# NDM-1-Positive *K. pneumoniae* at a Teaching Hospital in Southwestern China: Clinical Characteristics, Antimicrobial Resistance, Molecular Characterization, Biofilm Assay, and Virulence

**DOI:** 10.1155/2020/9091360

**Published:** 2020-10-09

**Authors:** Kai Yang, Shumin Liu, Huanqin Li, Na Du, Jing Yao, Qiuyue He, Yan Du

**Affiliations:** ^1^Department of Clinical Laboratory, The First Affiliated Hospital of Kunming Medical University, Kunming 650032, China; ^2^Yunnan Key Laboratory of Laboratory Medicine, Kunming 650032, China; ^3^Yunnan Innovation Team of Clinical Laboratory and Diagnosis, First Affiliated Hospital of Kunming Medical University, Kunming 650032, China; ^4^Department of Clinical Laboratory, The No. 1 Affiliated Hospital of Yunnan University of Chinese Medicine, Kunming, China

## Abstract

**Background:**

The emergence of the NDM-1-positive *Klebsiella pneumoniae* (*K. pneumoniae*) strains has led to limited therapeutic options for clinical treatment. Understanding the clinical characteristics, antimicrobial resistance, biofilm assay, and the virulence genes of these isolated strains is of great significance.

**Methods:**

The polymerase chain reaction (PCR) was used to screen isolated NDM-1-positive *K. pneumoniae*. The clinical information of the patients was collected from medical records. The NDM-1-positive *K. pneumoniae* isolates were subjected to antimicrobial susceptibility testing and multilocus sequence typing. Sixty strains of NDM-1-negative *K. pneumoniae* isolated during the same period were collected as the control group for the virulence analysis. The virulence phenotype of the strains was preliminarily evaluated by the string test and crystal violet semiquantitative biofilm formation experiment. PCR combined with gene sequencing was used to detect common high toxicity capsule genes (K1, K2, K5, K20, K54, and K57) and common virulence-related genes (*entB, ybtS, ureA, ycf, WabG, FimH, uge, iutA, KfuB, aerobactin, rmpA, magA, Alls, IrnN, and VatD*).

**Results:**

In the 30 nonduplicated NDM-1-positive *K. pneumoniae* isolates, 43.33% (13/30) of the patients had a history of a stay in the neonatal intensive care unit (NICU). All of the isolates exhibited multidrug resistance. Nine STs were identified, 77% (10/13) strains from the NICU were ST11. The NDM-1-positive *K. pneumoniae* string tests were all negative, and 35% (21/60) NDM-1-negative *K. pneumoniae* were positive. The ratios of NDM-1-positive *K. pneumoniae* isolates biofilm formation ability according to strong, medium, and weak classification were 67%, 23%, and 10%, respectively. NDM-1-negative *K. pneumoniae* isolates were 60%, 25%, and 15%, respectively. There was no statistical difference between the two groups (*t* = 0.61, *P*=0.2723). The virulence-associated genes with more than 80% of detection rates among the 30 NDM-1-positive *K. pneumoniae* isolates included *entB* (100%, 30/30), *ybtS* (93.33%, 28/30), *ureA* (90%, 27/30), *ycf* (83.33%, 25/30), and *wabG* (90%, 27/30). *KfuB* and *iutA* were detected at prevalence of 3.33% and 13.33%. *vatD, allS, iroN, aerobactin,* and *rmpA* were not detected. In the NDM-1-negative *K. pneumoniae*, all other 14 virulence genes except *VatD* were detected. After statistical analysis, *FimH, WabG, ycf, iutA, kfuB, aerobactin, rmpA,* and *Alls* virulence genes, *P* < 0.005, there was a statistical difference.

**Conclusion:**

NDM-1-positive *K. pneumoniae* exhibited multidrug resistance, MLST typing is mainly ST11, there is small clonal dissemination in the NICU in the hospital, and the NDM-1-positive *K. pneumoniae* virulence genes carrier rate is lower than the NDM-1-negative *K. pneumoniae* virulence genes carrier rate.

## 1. Introduction

As far as humans are concerned, *K. pneumoniae* is a frequently-isolated bacterial pathogen that colonizes the oropharynx, skin, or gastrointestinal tract. It causes various infections, including bacteraemia, pneumonia, urinary tract infections, suppurative infections, and cholangitis, especially to patients suffering from underlying disease conditions such as diabetes mellitus [1, 2].

The New Delhi metallo-*β*-lactamase (*NDM*) is a metallo-*β*-lactamase able to hydrolyze almost all *β*-lactams [3]. NDM-1 was first identified in a *K. pneumoniae* strain isolated from a Swedish patient who had been hospitalized in New Delhi, India, in 2008 [4]. NDM-positive strains cause a variety of infections that have been reported to be associated with high mortality rates [5]. NDM-positive strains have been found worldwide, resulting in a significant challenge for clinical management and public health [6]. Twenty-four NDM variants have been identified in more than 60 species of 11 bacterial families, and several variants have shown enhanced carbapenemase activity [7]. Among the 24 NDM variants, NDM-1 has the widest host spectrum identified so far and has been found in a number of species belonging to 11 bacterial families. *K. pneumoniae* and *Escherichia coli* are the predominant carriers of *blaNDM-1* [8].

Research has shown that *K. pneumoniae* with the NDM-1 genotype was the primary cause of neonatal carbapenem resistant sepsis in China [9]. Fuursted, et al. have reported that *K. pneumoniae* carrying NDM-1 have an intrinsic virulence potential [10]. Researchers also confirmed that bacteria can increase drug resistance through a variety of drug resistance mechanisms, while improving its pathogenicity [11].

Virulence factors are important in colonization, invasion, and development of infection, and the virulence factors such as lipopolysaccharides (LPS), capsule, siderophores, and fimbriae in *K. pneumoniae* have been well characterized to date [12].

Previous studies have focused on the risk factors of *blaNDM-1* and the surrounding environment of genes. Our previous studies have shown that *NDM-1* can be expressed on chromosomes and plasmids, and NDM-1-positive *K. pneumoniae is multidrug resistant* and can spread easily [13]. However, little is known about the virulence factor characteristics of NDM-1-positive *K. pneumoniae*. In this study, the clinical infection characteristics, drug resistance characteristics, and MLST homology typing of NDM-1-positive *K. pneumoniae* were analyzed, and NDM-1-negative *K. pneumoniae* was used as a control group. The phenotype and virulent genes were compared, and new ideas for the research on infection control, clinical treatment, and the pathogenicity of NDM-1-positive *K. pneumoniae* were proposed.

## 2. Materials and Methods

### 2.1. Collection and Identification of NDM-1-Positive *K. pneumoniae* Isolates

The 30 NDM-1-positive *K. pneumoniae* isolates were screened from 720 *K. pneumoniae* isolates at the First Affiliated Hospital of Kunming Medical University, in Yunnan Province, Southwest China, from January 2017 to June 2020. They were obtained from sputum (18/30), urine (5/30), catheter tip (3/30), blood (3/30), and wound (1/30). The isolates were identified as *K. pneumoniae* strains by using the VITEK-2 System (bioMe'rieux). Also, PCR was performed to detect *blaNDM-1* (Forward 5′-GGGCAGTCGCTTCCAACGGT-3′, Reverse 5′-GTAGTGCTCAGTGTCGGCAT-3′) [14]. Clinical information was collected, including demographics, underlying medical conditions, clinical presentations, and antimicrobial therapy. This study was approved, and informed consent was acquired from the patients involved in this study.

### 2.2. Antimicrobial Susceptibility Testing

The antimicrobial susceptibility testing was performed with a VITEK-2 automated microbiology analyzer platform (bioMérieux, Marcy l'Etoile, France) to examine the sensitivity of NDM-1-positive *K. pneumoniae* against common antibiotics. The minimal inhibitory concentration (MIC) of imipenem was further verified by the E-test method according to the guideline recommended by the Clinical and Laboratory Standards Institute (CLSI, 2018), and the MIC of colistin B was further verified by the microdilution broth method according to the guideline recommended by the European Committee on Antimicrobial Susceptibility Testing (EUCAST, 2018). *E. coli* ATCC25922 was used as a control strain for the antimicrobial susceptibility testing.

### 2.3. Multilocus Sequence Typing (MLST)

MLST was used to screen the 30 NDM-1-positive *K. pneumoniae* isolates by amplifying seven housekeeping genes (*gapA, infB, mdh, pgi, phoE, rpoB, and tonB*) expressed in *K. pneumoniae* according to the protocol at (http://bigsdb.pasteur.fr/klebsiella/primers_used.html).

### 2.4. String Test

*K. pneumoniae* strains were incubated overnight on blood agar. A single colony was touched with a loop and stretched outward. The length of the viscous string was pulled upward and measured. A positive string test result was defined as a string longer than 5 mm. The string test was repeated three times for each strain, and the final result was determined [15].

### 2.5. Biofilm Formation Assay

In brief, 10 *μ*l of the 0.5 McFarland bacterial standard and 190 *μ*l of the Luria-Bertani (LB) broth were inoculated into the wells of a 96-well microplate, with three wells per strain, and the microplate was incubated at 37 °C for 24 h. Thereafter, the LB broth was removed, and the bacterial cells were stained with 200 *μ*l of 0.1% crystal violet at room temperature for 15 min and, then, the due was removed. The plate was washed with distilled water and, then, dried. The absorbance was measured with a microplate reader set at 590 nm after adding 200 *μ*l of ethanol for 10 min into the wells. The yield of biofilm formation of the strains was interpreted as follows: OD > 0.5 as strong-producing, 0.2 ≤ OD ≤ 0.5 as moderate-producing, and OD < 0.2 as weak-producing [16].

### 2.6. Virulence-Associated Genes

The primer sequences for capsular serotyping and the virulence genes are listed in Supplementary Materials. Capsular serotypes, including K1, K2, K5, K20, K54, and K57, were determined using the methods described previously [17, 18]. The fifteen virulence-associated genes, including *aerobactin*, *iroN*, *kfuB*, *rmpA*, *alls*, *ybtS*, *ureA*, *uge*, *wabG*, *ycf*, *entB*, *iutA*, *aerobactin, vatD*, *magA,* and *fimH,* were determined by PCR using the primers described previously [19].

### 2.7. Statistical Analysis

All statistical analyses were performed using SPSS 22.0 software (IBM, Armonk, NY, USA). The categorical variables were listed as percentages and evaluated using the Chi-square test or Fisher's exact test. The continuous data were expressed as mean ± standard deviation (mean ± SD) or median (25th-75th percentile) appropriately and analyzed using Student's *t*-test or the Mann–Whitney *U* test. A *P* value <0.05 was considered statistically significant. All tests were two-tailed.

## 3. Results

### 3.1. Clinical Characteristics of Patients with NDM-1-Positive *K. pneumoniae*

Clinical characteristics of 30 patients with NDM-1-positive *K. pneumoniae* isolates are shown in Table 1. In the 30 nonduplicated NDM-1-positive *K. pneumoniae* isolates, the patients' median length of hospitalization was 49 days. 43.3% (13/30) of the patients had a history of a stay in the neonatal intensive care unit (NICU). Of these patients, 63.3% (19/30) were males. All, but five patients, received invasive treatment prior to infection with NDM-1-positive *K. pneumoniae*, including central venous catheters and invasive mechanical ventilators. 70% (21/30) had been treated by carbapenems (imipenem and meropenem).

### 3.2. Antimicrobial Susceptibility Testing

The antimicrobial resistance rates of the 30 NDM-1-positive *K. pneumoniae* isolates are shown in Table 2. They were all resistant to meropenem, imipenem, ertapenem, ceftazidime, cefoperazone sulbactam, piperacillin, sulbactam, cefazolin, cefepime, and cefoxitin. Their minimal inhibitory concentrations (MICs) of imipenem were more than 4 *μ*g/ml by the E-test method. Twenty-six (86.67%, 26/30) isolates were resistant to aztreonam and ciprofloxacin, and twenty-three (76.7%, 23/30) were resistant to levofloxacin. Twenty-two (73.3%, 22/30) and seventeen (56.7%, 17/30) isolates were resistant to gentamicin and amikacin, respectively. Only ten isolates were resistant to sulfamethoxazole/trimethoprim, and all isolates were susceptible to colistin B and tigecycline.

### 3.3. Molecular Characteristics of NDM-1-Positive *K. pneumoniae* Isolates

**Figure 1:**
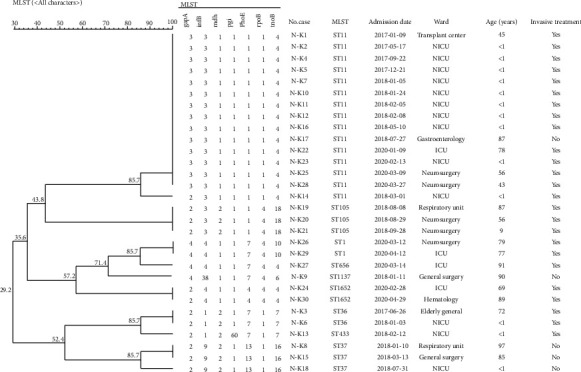
The specific sequence similarity cluster analysis.

Among the 30 NDM-1-positive *K. pneumoniae* isolates, 9 STs were identified, including ST11 (15 isolates), ST105 (3 isolates), ST37(3 isolates), ST1 (2 isolates), and ST36 (2 isolates), ST1652 (2 isolates), ST656 (1 isolate), ST1137 (1 isolate), and ST433 (1 isolate). Ten of the thirteen strains from the NICU were ST11. Others were ST433, ST36 and ST37, respectively. The specific sequence similarity cluster analysis is shown in Figure 1.

### 3.4. String Test

String test results of NDM-1-positive *K. pneumoniae* isolates were all negative, and 35% (21/60) NDM-1-negative *K. pneumoniae* isolates were positive.

### 3.5. Biofilm-Producing Isolates

**Figure 2:**
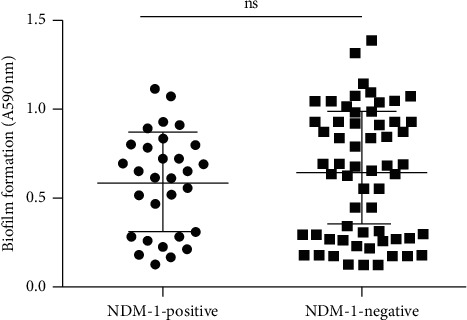
The biofilm formation. *Note.* The circles indicate the 30 NDM-1-positive *K. pneumoniae*; the squares indicate the 60 NDM-1-negative *K. pneumoniae*.

The biofilm-producing analysis is shown in Figure 2. The ratios of NDM-1-positive *K. pneumoniae* isolates biofilm formation ability according to strong, medium, and weak classification were 67%, 23%, and 10%, respectively. NDM-1-negative *K. pneumoniae* isolates were 60%, 25%, and 15%, respectively. The biofilm-forming capacity of NDM-1-positive *K. pneumoniae* isolates and NDM-1-negative *K. pneumoniae* isolates measured A590 were 0.5767 ± 0.2854 and 0.6225 ± 0.3595, respectively. There was no statistical difference between the two groups (*t* = 0.61, *P*=0.2723).

### 3.6. Prevalence of Capsular Serotyping and Virulence-Associated Genes

The 6 serotypes and 15 virulence genes were tested in this experiment. However, we did not detect any serotypes in NDM-1-positive *K. pneumoniae* strains. Among the 60 NDM-1-negative *K. pneumoniae* isolates, 15 (25%) isolates belonged to capsular serotype K1, 7(11.6%) isolates belonged to capsular serotype K2, 1 (1.6%) isolate belonged to capsular serotype K5, and 2 (3.33%) isolates belonged to capsular serotype K57. The remaining 35 (58.3%) isolates were not successfully typed. The prevalence and distribution of virulence factors are shown in Table 3. The virulence-associated genes with more than 80% of detection rates among the 30 isolates included *entB* (100%, 30/30), *ybtS* (93.33%, 28/30), *ureA* (90%, 27/30), *ycf* (83.33%, 25/30), and *wabG* (90%, 27/30). *KfuB* and *iutA* were detected at a prevalence of 3.33% and 13.33%, respectively. *vatD, allS, iroN, aerobactin,* and *rmpA* were not detected in NDM-1-positive *K. pneumonia*e isolates. The NDM-1-positive *K. pneumoniae* group carried 6 or 7 virulence genes most commonly, and the NDM-1-negative *K. pneumoniae* group mainly carried 8–11 virulence genes. In NDM-1-negative *K. pneumoniae*, all other 14 virulence genes except *VatD* were detected. After statistical analysis, *FimH, WabG, ycf, iutA, kfuB, aerobactin, rmpA,* and *Alls* virulence genes, *P* < 0.005, showed there was a statistical difference.

## 4. Discussion

*K. pneumoniae* causes a wide range of infections both in the community and health-care setting leading to increased morbidity and mortality [20]. The patients infected with NDM-1-positive *K. pneumoniae* in this study have a large age span, mainly from newborns and elderly patients, suggesting that people with poor immunity and underlying diseases are susceptible to it. Ten of the 13 neonatal patients are premature and low birth weight infants, which may be related to their immune insufficiency, low phagocytic ability of white blood cell, and underdeveloped skin barrier. In addition, the 25 of 30 patients underwent invasive examinations, indicating that device intervention may weaken the patient's immunity. The specimens in this study were mainly derived from the sputum, suggesting that NDM-1-positive *K. pneumoniae* is more likely to spread through the respiratory tract. The sputum specimens are likely to cause environmental pollution and the spread of contact, so they should to be treated with strict disinfection measures to prevent its spread in the hospital.

The 30 patients had injected antibiotics before we separated the NDM-1-positive *K. pneumoniae* strains from them. Antibiotics are mainly cephalosporins antibiotics and penicillium carbon alkene. An in vitro susceptibility test showed that it was highly resistant to *β*-lactam drugs and *β*-lactamase inhibitor, but sensitive to aminoglycoside and fluoroquinolone, which is consisted with the literature that aminoglycoside is still recognized as a first-line therapy for treatment of *K. pneumoniae* infection [21]. In theory, strains carrying *blaNDM-1* should be sensitive to aztreonam, but drug susceptibility results showed that treatment with aztreonam alone had a poor curative effect. Except for polymyxin B and tigecycline, it was highly resistant to a majority of clinical antibiotics. It is because that *blaNDM-1* is mainly disseminated by plasmid IncA/C, which always carries a variety of resistance genes resulting in the emergence of antibiotics resistance [5]. Although aztreonam is stable against MBLs, NDM-1-positive *K. pneumoniae* strains usually have ESBLs or AmpC enzymes that are able to hydrolyze aztreonam. Aztreonam alone, therefore, has limited clinical utility against NDM-producing strains. In this study, all strains are sensitive to tigecycline and polymyxin B, which is consistent with Darey's research showing that polymyxin alone can treat infections caused by *NDM-*1-positive bacteria [22]. The use of colistin has also been hampered by the neurological adverse effects and occurrence of renal toxicity [23, 24]. There is evidence that polymyxin-based combinations may be more effective than polymyxin alone, so it is usually recommended to combine polymyxin with other antibiotics [25].

The 30 NDM-1-positive *K. pneumoniae* strains in this study were typed by MLST, and there were 9 types in total, which mainly is ST11. In China, most of the NDM-1-positive strains have been found belonging to different ST types and being scattered. ST11, ST14, ST15, and ST147 strains are relatively common NDM-positive *K. pneumoniae* lineages and have been found in multiple countries across several continents, almost all of which were isolated from humans [7]. In medical institutions and wards, if more than 3 cases of homogenous nosocomial infections occur in a short time, it is called nosocomial infection outbreak. In terms of time, the NO.10 strain was the first screened strain in the NICU ward. The patient was admitted to the hospital in January 24, 2018, and stayed for 20 days, which is overlapped with the 11^th^, 12^th^, and 13^th^ strains at the same time. Afterwards, the 14^th^ and 16^th^ strains were overlapped with the 12^th^ strain again. These 6 strains were genotyped by MLST, which shows 5 strains of them are ST11. Considering that the *NDM-1* is located in the plasmid and easy to spread, it suggests that there is a clonal spread of ST11 NDM-1-positive *K. pneumoniae* strains in the NICU.

At present, most studies define the positive strain in the string test as hvKP. In this experiment, NDM-1-positive *K. pneumoniae* string tests were all negative, while the positive rate of the string test in the NDM-1-negative group was as high as 53.3%. It preliminarily indicates that NDM-1-negative *K. pneumoniae* may be more virulent. However, some scholars believe that the string test cannot be used as a criterion for judging whether the *K. pneumoniae* has high virulence.

Biofilm can enhance the defense ability of bacteria and resist the killing effect of antibiotics. The results of this study showed that all strains formed biofilms at varying degrees. Although NDM-1-positive *K. pneumoniae* does not show enhanced biofilm forming ability, the formation of biofilm will prolong the disease, so we still need to be vigilant.

Capsular polysaccharide (CPS), as one of the most important virulence factors of *K. pneumoniae*, can resist the phagocytosis of macrophages and neutrophils. Among them, K1, K2, K5, K20, K54, and K57 are recognized as highly virulent serotype [12]. In this test, NDM-1-positive *K. pneumoniae* did not detect the abovementioned 6 capsular serotypes. This indicates that when the bacteria acquires drug resistance, the gene expression ability of common high-virulence capsule serotypes will be weakened, and it is also possible that resistant bacteria express other capsule serotypes that have not been detected. The 4 of 6 types of NDM-1-negative *K. pneumoniae* high-virulence capsular serotypes were detected, mainly K1 and K2. In this experiment, *magA* and *rmpA* were not detected in the NDM-1-positive *K. pneumoniae* group. 15% of NDM-1-negative *K. pneumoniae* carry *rmpA*, and 12% carry *magA*, which is basically consistent with the drawing experiment and the detection rate of high-virulence capsule serotypes. The positive rate in the NDM-1-negative group is higher than that in the control group. There are many virulence factors related to pathogenicity in *K. pneumoniae*. The basis of them is mainly the capsular polysaccharide and iron uptake system. In addition, it also includes high mucus, lipopolysaccharide, and fimbriae-related (type I fimbriae and type III Pili), and biofilm formation. The 30 strains of NDM-1-positive *K. pneumoniae* are most widely distributed in *entB, ureA, ybtS, FimH, uge,* and *ycf.* These virulence genes are related to bacterial adhesion, iron uptake, and anti-phagocytosis. Although the positive rate of NDM-1-positive *K. pneumoniae* in the drawing experiment and common capsular serotypes is zero, it still expresses many important virulence genes.

Once *K. pneumoniae* inserts the virulence gene into the drug-resistant plasmid, it will make it a highly resistant, highly virulent, and easily spread strain. Some literatures have reported that highly virulent and resistant strains have begun to appear all over the world. In 2016, a case of *blaNDM-1*-positive ST231 CR-hvKP was reported in India. Also, a case of *blaNDM-1*-positive ST23 type CR-hvKP was reported in Europe in 2017 [26]. There were also several cases of CR-hvKP carrying *blaNDM-1* reported in China [27, 28].

In summary, compared with the control group, the positive rate of NDM-1-positive *K. pneumoniae* in the string test and common high-virulence capsular serotypes, as well as the distribution of these virulence genes, are significantly reduced, which is consistent with Montanari's claim that bacteria lose some virulence genes in order to obtain resistance genes for optimal adaptability [29]. It indicates that although NDM-1-positive *K. pneumoniae* has increased drug resistance, its pathogenicity may be weaker. However, the NDM-1-positive *K. pneumoniae* still expresses many important virulence factors.

## Figures and Tables

**Table 1:** Clinical characteristics of patients with NDM-1-positive *K. pneumoniae*.

Isolate no.	Gender	Age (yr)	Date of specimen collection	Days in the hospital	Isolation site(s)	Ward	Invasive treatment	Treatment	Outcome
K-N1	Male	45	2017/1/9	89	Sputum	Transplant center	Laparotomy	SCF, MEM, TEC	Recovered
K-N2	Female	Newborn	2017/5/17	39	Sputum	NICU	Umbilical vein cannula	AMC, SCF, FLUCZ, AZT, CRO	Recovered
K-N3	Male	72	2017/6/26	36	Sputum	Elderly general	Tracheal intubation	MEM, KETOC, VA	Died
K-N4	Male	Newborn	2017/9/22	46	Catheter tip	NICU	Umbilical vein intubation; central venous catheterization	SCF, AZT, FLUCZ, TZP, CAZ	Recovered
K-N5	Male	Newborn	2017/12/21	84	Sputum	NICU	Noninvasive ventilator	MEM, AMC, PIS	Recovered
K-N6	Female	Newborn	2018/1/3	10	Sputum	NICU	Tracheal intubation; umbilical vein catheter	AMC, SCF	Died
K-N7	Male	Newborn	2018/1/5	28	Urine	NICU	Umbilical vein catheterization; ventilator oxygen	AMC, FLUCZ	Recovered
K-N8	Male	97	2018/1/10	13	Sputum	Respiratory unit	No	MEM, KETOC, LEV	Recovered
K-N9	Male	90	2018/1/11	20	Sputum	General surgery	No	MEM, FLUCZ	Died
K-N10	Female	Newborn	2018/1/24	20	Urine	NICU	Tracheal intubation; umbilical vein catheterization	MEM, AMC, SCF	Recovered
K-N11	Male	Newborn	2018/2/5	33	Catheter tip	NICU	Tracheal intubation; umbilical vein catheterization	MEM, AMC	Recovered
K-N12	Female	Newborn	2018/2/8	100	Sputum	NICU	Tracheal intubation; umbilical vein catheterization	AMC	Died
K-N13	Male	Newborn	2018/2/12	16	Sputum	NICU	Tracheal intubation	SCF	Recovered
K-N14	Female	Newborn	2018/3/1	51	Urine	NICU	Umbilical vein catheterization; noninvasive ventilator	MEM, AMC, SCF	Recovered
K-N15	Male	85	2018/3/13	27	Sputum	General surgery	No	MOX	Recovered
K-N16	Female	Newborn	2018/5/10	64	Catheter tip	NICU	Tracheal intubation; umbilical vein catheterization	MEM, SCF, IPM	Recovered
K-N17	Male	86	2018/7/27	14	Sputum	Gastroenterology	No	MEM	Died
K-N18	Female	87	2018/7/31	9	Sputum	NICU	No	SCF	Recovered
K-N19	Male	87	2018/8/8	14	Sputum	Respiratory unit	Noninvasive ventilator	MEM, FLUCZ, SCF	Died
K-N20	Male	56	2018/8/29	14	Blood	Neurosurgery	Tracheal intubation, noninvasive ventilator;	MEM, VA	Recovered
K-N21	Male	9	2018/9/28	91	Blood	Neurosurgery	Craniopharyngioma resection; lateral ventricle puncture	MEM, CZO, VA	Recovered
K-N22	Female	49	2020/1/9	56	Blood	ICU	Tracheal intubation	TZP, IMP	Recovered
K-N23	Male	82	2020/2/13	18	Sputum	NICU	Tracheal intubation	CMZ, TZP, IMP	Recovered
K-N24	Female	47	2020/2/28	13	Urine	ICU	Tracheal intubation	LVX, IMP, SCF, MEM	Recovered
K-N25	Male	91	2020/3/9	18	Sputum	Neurosurgery	No	IMP, Cefoselis, LVX	Recovered
K-N26	Male	88	2020/3/12	15	Sputum	Neurosurgery	Tracheal intubation	MEM, TZP, LVX	Recovered
K-N27	Male	62	2020/3/14	25	Urine	ICU	Tracheal intubation	MEM, TZP	Recovered
K-N28	Female	59	2020/3/27	14	Sputum	Neurosurgery	Lateral ventricle puncture	TZP, MXF	Recovered
K-N29	Female	93	2020/4/12	46	Wound	ICU	Tracheal intubation	MEM, TZP, SCF, LVX	Recovered
K-N30	Male	14	2020/4/29	21	Sputum	Hematology	Tracheal intubation	MEM, TZP	Recovered

*Notes*. SCF: cefoperazone sulbactam; TEC: teicoplanin; MEM: meropenem; CRO: ceftriaxone; MOX: Lafaxed; AMC: amoxicillin clavulanate potassium; KETOC: voriconazole; FLUCZ: fluconazole; AZT: aztreonam; VA: vancomycin; CAZ: ceftazidime.

**Table 2:** The antimicrobial resistance profiling of NDM-1-positive *K. pneumoniae*

	NDM-1-positive *K. pneumoniae* (*n* = 30)	
	No.	%
Meropenem	30	100%
Imipenem	30	100%
Ertapenem	30	100%
Ceftazidime	30	100%
Cefoperazone sulbactam	30	100%
Piperacillin and sulbactam	30	100%
Cefazolin	30	100%
Cefepime	30	100%
Cefoxitin	30	100%
Ciprofloxacin	26	86.67%
Aztreonam	26	86.67%
Levofloxacin	23	76.67%
Gentamicin	22	73.33%
Amikacin	17	56.67%
Sulfamethoxazole/trimethoprim	10	33.30%
Polymyxin B	0	0
Tigecycline	0	0

**Table 3:** Differences in the distribution of virulence genes and serotypes between NDM-1-positive *K. pneumoniae* and NDM-1-negative *K. pneumonia* (n (%)).

	NDM-1-positive *K. pneumoniae* (*n* = 30)	NDM-1 negative *K. pneumoniae* (*n* = 60)	*P*
*K1*	0 (0)	15 (25.00)	0.003
*K2*	0 (0)	7 (11.66)	0.051
*K5*	0 (0)	1 (1.66)	0.477
*K57*	0 (0)	2 (3.33)	0.312
*entB*	30 (100.00)	60 (100.00)	1
*ybtS*	28 (93.33)	60 (100.00)	0.109
*FimH*	23 (76.67)	56 (93.33)	0.038
*wabG*	27 (90.00)	60 (100.00)	0.035
*ureA*	27 (90.00)	59 (98.33)	0.106
*ycf*	25 (83.33)	58 (96.67)	0.039
*uge*	23 (76.67)	43 (71.67)	0.801
*iutA*	41 (3.33)	26 (43.33)	0.005
*kfuB*	1 (3.33)	20 (33.33)	0.001
*aerobactin*	0 (0)	13 (21.67)	0.004
*IroN*	0 (0)	1 (1.67)	1
*rmpA*	0 (0)	9 (15.00)	0.027
*magA*	0 (0)	7 (11.67)	0.09
*Alls*	0 (0)	9 (15.00)	0.027
vatD	0 (0)	0 (0)	1

## Data Availability

All data that were used to support the findings of this study are included within the article.

## Supplementary Materials

Supplementary Materials The sequences of primers for capsular serotyping and virulence-associated genes.
