# Supplementary material for: NDM-1-Positive K. pneumoniae at a Teaching Hospital in Southwestern China: Clinical Characteristics, Antimicrobial Resistance, Molecular Characterization, Biofilm Assay, and Virulence
Source: Can J Infect Dis Med Microbiol. 2020 Oct 9;2020:9091360. doi: 10.1155/2020/9091360 (PMC7568808; doi:10.1155/2020/9091360)
Supplement: Supplementary Materials — The sequences of primers for capsular serotyping and virulence-associated genes. [file 9091360.f1.docx]

**Supplemental materials**

The sequences of primers for capsular serotyping and virulence-associated genes

| Genes | Sequence (5′ →3′) | T（℃） | Fragment（bp） |
| --- | --- | --- | --- |
| K1 | F: GGTGCTCTTTACATCATTGC  R: GCAATGGCCATTTGCGTTAG | 54 | 1283 |
| K2 | F: GACCCGATATTCATACTTGACAGAG  R: CCTGAAGTAAAATCGTAAATAGATGGC | 64 | 641 |
| K5 | F: TGGTAGTGATGCTCGCGA  R: CCTGAACCCACCCCAATC | 64 | 280 |
| K20 | F: CGGTGCTACAGTGCATCATT  R: GTTATACGATGCTCAGTCGC | 56 | 741 |
| K54 | F: CATTAGCTCAGTGGTTGGCT  R: GCTTGACAAACACCATAGCAG | 62 | 881 |
| K57 | F: CTCAGGGCTAGAAGTGTCAT  R: CACTAACCCAGAAAGTCGAG | 59 | 1037 |
| ureA | F: GACAAGCTGTTGCTGTTTACC  R: CGGGTTGTGAACGGTGAC | 58 | 270 |
| wabG | F: ACCATCGGCCATTTGATAGA  R: CGGACTGGCAGATCCATATC | 58 | 683 |
| fimH | F: TGCTGCTGGGCTGGTCGATG  R: GGGAGGGTGACGGTGACATC | 62 | 909 |
| *entB* | F: ATTTCCTCAACTTCTGGGGC  R: AGCATCGGTGGCGGTGGTCA | 56 | 371 |
| ycf | F: ATCAGCAGTCGGGTCAGC  R: CTTCTCCAGCATTCAGCG | 58 | 160 |
| *ybtS* | F: CACCGCAAACGCAATCTG  R: GCCATAGACGCTGTTGTTGA | 56 | 782 |
| iutA | F: GGCTGGACATCATGGGAACTGG  R: CGTCGGGAACGGGTAGAATCG | 66 | 300 |
| rmpA | F: ACTGGGCTACCTCTGCTTCA  R: CTTGCATGAGCCATCTTTCA | 58 | 516 |
| aerobactin | F: GCATAGGCGGATACGAACAT  R: CACAGGGCAATTGCTTACCT | 58 | 556 |
| IroN | F: GGCTACTGATACTTGACTATTC  R: CAGGATACAATAGCCCATAG | 58 | 992 |
| KfuB | F: GAAGTGACGCTGTTTCTGGC  R: TTTCGTGTGGCCAGTGACTC | 58 | 960 |
| *wcaG* | F: GGTTGGKTCAGCAATCGTA  R: ACTATTCCGCCAACTTTTGC | 54 | 169 |
| alls | F: CCGAAACATTACGCACCTTT  R: ATCACGAAGAGCCAGGTCAC | 58 | 508 |
| uge | F: TCTTCACGCCTTCCTTCACT  R: GATCATCCGGTCTCCCTGTA | 60 | 534 |
| vatD | F: GTTTTATTTCGTTAGCAG  R: GAAGGAAACAAATCAGTA | 48 | 463 |
